# PAQR3 Regulates Endoplasmic Reticulum-to-Golgi Trafficking of COPII Vesicle via Interaction with Sec13/Sec31 Coat Proteins

**DOI:** 10.1016/j.isci.2018.11.002

**Published:** 2018-11-04

**Authors:** Qianqian Cao, Zheng Wang, Huida Wan, Lijiao Xu, Xue You, Lujian Liao, Yan Chen

**Affiliations:** 1 CAS Key Laboratory of Nutrition, Metabolism and Food Safety, Shanghai Institute of Nutrition and Health, Shanghai Institutes for Biological Sciences, University of Chinese Academy of Sciences, Chinese Academy of Sciences, Shanghai 200031, China; 2 Shanghai Key Laboratory of Regulatory Biology, School of Life Sciences, East China Normal University, Shanghai 200241, China; 3 School of Life Sciences and Technology, Shanghai Tech University, Shanghai 200031, China

**Keywords:** Molecular Biology Experimental Approach, Cell Biology, Functional Aspects of Cell Biology

## Abstract

Endoplasmic reticulum (ER)-to-Golgi anterograde transport is driven by COPII vesicles mainly composed of a Sec23/Sec24 inner shell and a Sec13/Sec31 outer cage. How COPII vesicles are tethered to the Golgi is not completely understood. We demonstrated here that PAQR3 can facilitate tethering of COPII vesicles to the Golgi. Proximity labeling using PAQR3 fused with APEX2 identified that many proteins involved in intracellular transport are in close proximity to PAQR3. ER-to-Golgi trafficking of N-acetylgalactosaminyltransferase-2 on removal of brefeldin A is delayed by PAQR3 deletion. RUSH assay also revealed that ER-to-Golgi trafficking is affected by PAQR3. The N-terminal end of PAQR3 can interact with the WD domains of Sec13 and Sec31A. PAQR3 enhances Golgi localization of Sec13 and Sec31A. Furthermore, PAQR3 is localized in the ERGIC and cis-Golgi structures, the acceptor sites for COPII vesicles. Taken together, our study uncovers a role for PAQR3 as a player in regulating ER-to-Golgi transport of COPII vesicles.

## Highlights

• ER-to-Golgi trafficking of COPII vesicles is affected by PAQR3
• PAQR3 is localized in the ERGIC and cis-Golgi structures
• PAQR3 interacts with the coat proteins Sec13 and Sec31 of COPII vesicles
• PAQR3 facilitates tethering of COPII vesicles to the Golgi apparatus

## Graphical Abstract

**Figure:**
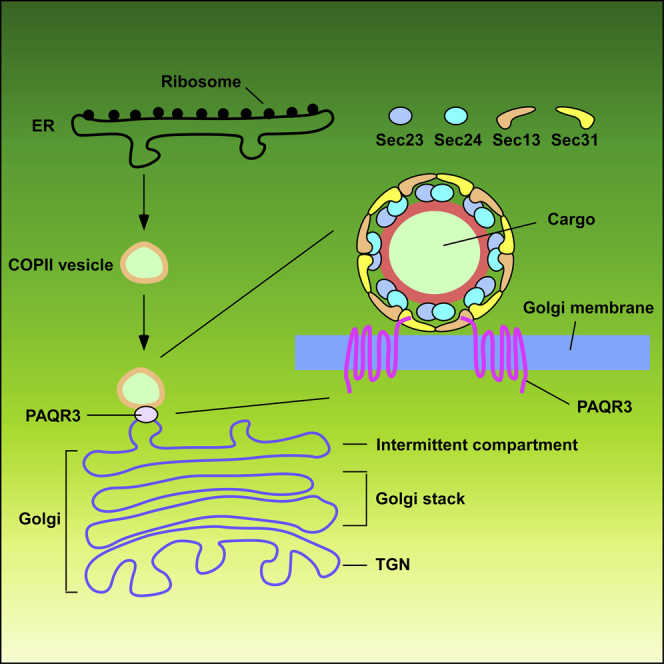

## Introduction

Cellular homeostasis relies on the coordinated interactions of proteins and the correct localization of those proteins to specific subcellular compartments. Mapping these network and subcellular proteome associated with a protein is critical for revealing its biological functions. There have been several experimental approaches established to globally define protein interactions and localization. Proximity labeling is one of the most effective approaches that can obtain spatially resolved proteomic maps of specific proteins and compartments within living cells (Hung et al., 2016). Proximity labeling relies on a engineered ascorbate peroxidase (APEX), which is capable of generating free biotinyl radicals to enable a rapid and spatially restricted labeling of proteins in the vicinity of the enzyme (Bersuker et al., 2018, Kim and Roux, 2016, Mick et al., 2015). APEX is a first-generation enzyme with triple mutations, whereas APEX2 is the improved second-generation enzyme with one additional mutation (Jiang et al., 2011, Lam et al., 2015, Martell et al., 2012, Rhee et al., 2013, Zhang et al., 2010). The irreversible conjugation of biotin catalyzed by APEX2 enables the capture of labeled proteins for proteomic analysis.

PAQR3, also named as RKTG for Raf kinase trapping to Golgi, is a Golgi-resident seven-transmembrane protein and plays a key role in maintaining cellular and physiological homeostasis (Feng et al., 2007, Luo et al., 2008). As a member of the progestin and AdipoQ receptor (PAQR) family, PAQR3 is conserved during evolution (Tang et al., 2005b). Previous studies have demonstrated that PAQR3 is a multifunctional protein. PAQR3 suppresses cell growth and tumorigenesis mainly through negative regulation of Ras-Raf-MEK-ERK and PI3K-AKT signaling pathways (Feng et al., 2007, Jiang et al., 2010, Wang et al., 2013). Both human and mouse studies have indicated that PAQR3 is a tumor suppressor that has an inhibitory function in many types of tumors (Jiang et al., 2011, Ling et al., 2014, Wang et al., 2012, Xie et al., 2008, Zhang et al., 2010). PAQR3 can regulate Golgi-to-plasma membrane (PM) transport via the Gβγ–PKD signaling pathway (Hewavitharana and Wedegaertner, 2015). Additionally, PAQR3 can control autophagy on glucose and amino acids starvation by integrating AMPK signaling to improve ATG14L-associated class III PI3K activity and by modulating mTOC1 activity (Wang et al., 2017, Xu et al., 2016). PAQR3 also plays a critical role in DNA damage repair by affecting the function of RAD23B-XPC (You et al., 2017). Moreover, PAQR3 has an important effect on hepatic lipid catabolism by promoting PPARα ubiquitination through the E3 ubiquitin ligase HUWE1 (Zhao et al., 2018).

In this study, we applied the proximity labeling strategy to further investigate the interaction networks of PAQR3. We constructed APEX2-fused PAQR3 to label and capture proteins adjacent to PAQR3 in live cells. We then characterized the captured proteins by mass spectrum analysis. A series of detailed analyses led us to discover that PAQR3 can regulate ER-to-Golgi transport through interacting with COPII components Sec13/Sec31A, thereby extending the understanding about the biological functions of PAQR3, a key protein in maintaining cellular homeostasis.

## Results

### APEX2 Can Be Used to Selectively Biotinylate Proteins in Close Proximity to PAQR3

**Figure 1:**
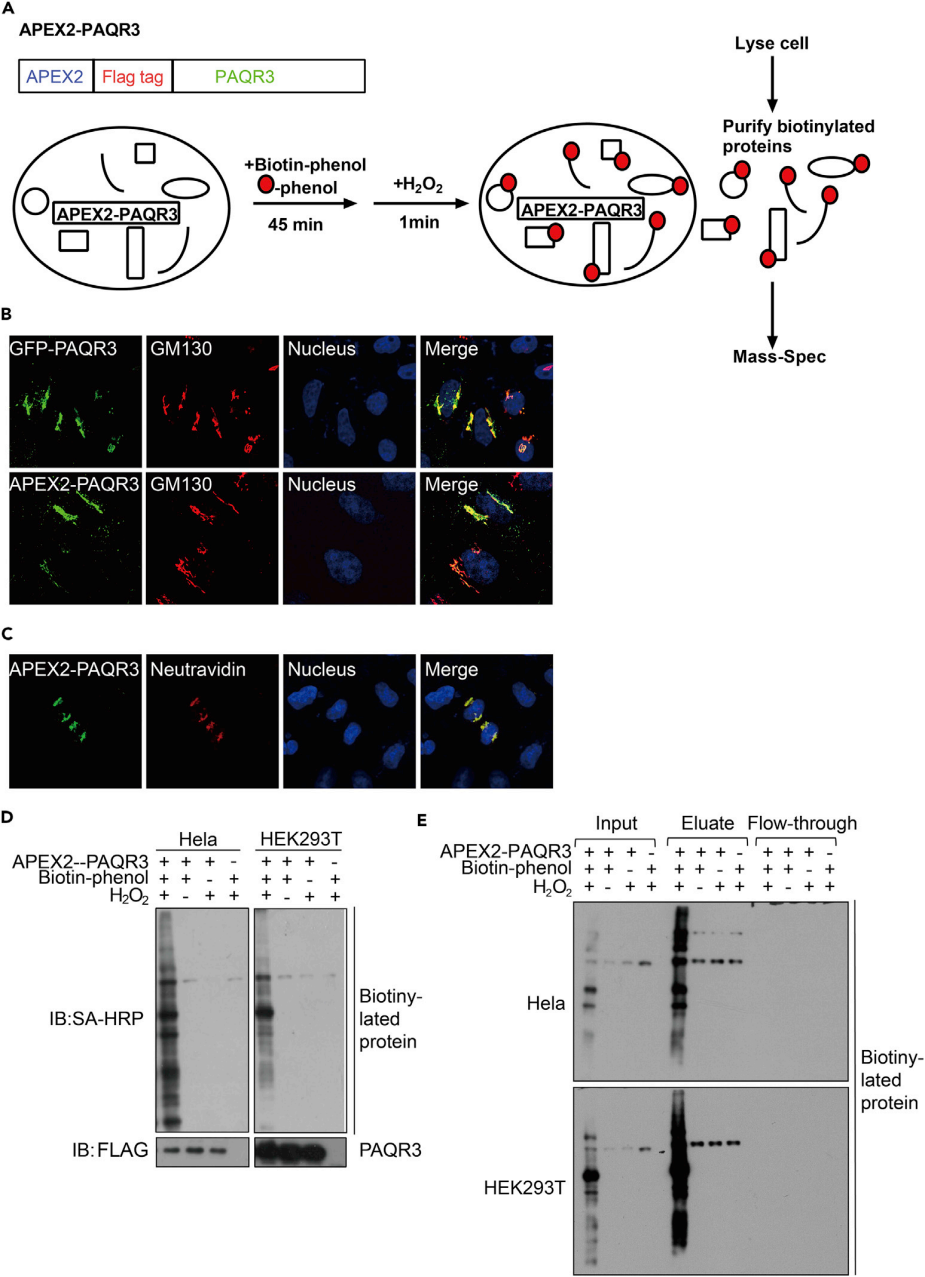
Establishment of Proximity Labeling Using APEX2-PAQR3 Fusion Protein (A) Diagram of the APEX2-PAQR3 labeling strategy. APEX2 and a FLAG tag was fused to the N terminus of PAQR3. On sequential treatment with biotin-phenol and H_2_O_2_, proteins in close spatial proximity with PAQR3 would be biotinylated (shown as red dots) and then subjected to mass spectrometry analysis. (B) Subcellular distribution of APEX2-fused PAQR3. HeLa cells were transiently transfected with FLAG-tagged APEX2-PAQR3 or GFP-fused PAQR3. The APEX2-PAQR3 was detected by an antibody against FLAG, followed by immunofluorescent staining. The Golgi was stained with an antibody against GM130, and the nucleus was stained with Hoechst 33342. (C) Subcellular distribution of biotinylated proteins. HeLa cells expressing APEX2-PAQR3 were labeled as in (A) and were stained with a NeutrAvidin-Alexa Fluor 647 (AF647) conjugate to visualize biotinylated proteins. An anti-FLAG antibody was used to visualize APEX-PAQR3. (D) Characterization of APEX-PAQR3-mediated biotinylation of endogenous proteins. Hela and HEK293T cells were transfected with APEX2-PAQR3 and treated with or without biotin-phenol/H_2_O_2_. The cell lysate was analyzed by immunoblotting with streptavidin-horseradish peroxidase (SA-HRP). The analysis was repeated three times with similar results. (E) Purification of biotinylated proteins. The proteins as in D were purified by streptavidin chromatography, and the samples were analyzed by immunoblotting with SA-HRP.

As a newly discovered tumor suppressor, PAQR3 is a protein that modulates various cellular functions (Feng et al., 2007, Hewavitharana and Wedegaertner, 2015, Jiang et al., 2010, Liu et al., 2015, Wang et al., 2013, Wang et al., 2017, Xu et al., 2015, Xu et al., 2016). To further investigate the subcellular microenvironment surrounding PAQR3 protein, we applied an APEX-based technology to characterize proteins that are in close proximity with the target protein (Hung et al., 2014, Hung et al., 2016, Lam et al., 2015). An ascorbate peroxidase 2 gene (APEX2) was fused with PAQR3 (Figure 1A). We first used immunofluorescence staining to analyze the subcellular localization of APEX2-PAQR3. As reported by previous studies (Feng et al., 2007, Luo et al., 2008), APEX2-PAQR3 was mainly localized in the Golgi apparatus (Figure 1B). We next characterized the enzymatic activity of APEX2-PAQR3 fusion protein. In the presence of H_2_O_2_, APEX can catalyze the conversion of biotin-phenol into biotin-phenoxyl radicals, which are short lived (<5 ms), are membrane impermeable, and have a small labeling radius (<20 nm) (Bendayan, 2001, Hung et al., 2014, Rhee et al., 2013). We incubated APEX2-PAQR3-expressing HeLa cells with biotin-phenol for 30 min, followed by treatment with H_2_O_2_ for 1 min. Under these conditions, staining of HeLa cells with NeutrAvidin-Alexa Fluor conjugate revealed positive biotinylated signals in the Golgi apparatus (Figure 1C), confirming that APEX2-PAQR3 could biotinylate neighboring proteins around the Golgi. We next analyzed the biotinylated proteins with immunoblotting analysis. Biotinylated proteins could be clearly detected in the presence of biotin-phenol and H_2_O_2_ in both Hela and HEK293T cells (Figure 1D). However, biotin-phenol or H_2_O_2_ alone could not enrich biotinylated proteins in both of these cells (Figures 1D and 1E). Collectively, these data indicate that the APEX technology can be successfully used to characterize proteins in close proximity to PAQR3 that is mainly localized in the Golgi apparatus.

### Identification of Proteins in Close Proximity with PAQR3

**Figure 2:**
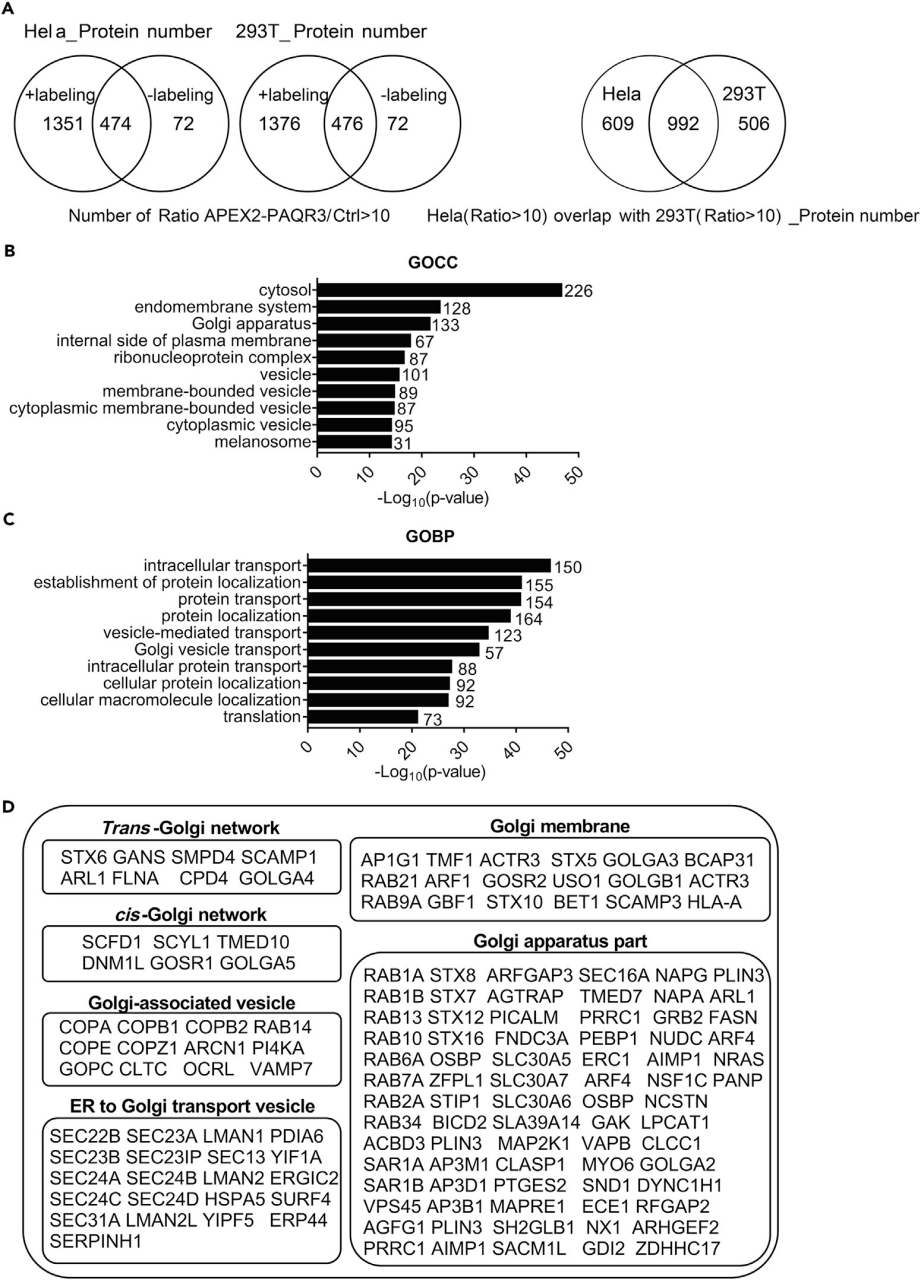
Identification of Proteins from Proximity Labeling with APEX2-PAQR3 (A) Venn diagram showing identified proteins after streptavidin chromatography from APEX labeling (+labeling) or mock labeling (without H_2_O_2_, -labeling) in HeLa cells and HEK293T cells. The right panel shows the proteins found in both cell types. The experiment was repeated three times with similar results. (B) Going analysis of cellular compartment (GOCC) of the 992 candidate proteins found in both HeLa and HEK293K cells. (C) Going analysis of biological process (GOBP) of the 992 candidate proteins found in both cell types. (D) A list of Golgi- and trafficking-associated proteins labeled by APEX2-PAQR3 in both cell types.

We next used streptavidin chromatography to isolate the biotinylated proteins in both Hela and HEK293T cells. As expected, eluting the biotinylated proteins from APEX2-PAQR3-expressing cells could successfully enrich the proteins biotinylated by APEX2 in the presence of biotin-phenol and H_2_O_2_ (Figure 1E). As negative controls, biotin-phenol or H_2_O_2_ alone could not give rise to biotinylated proteins (Figure 1E). We applied liquid chromatography (LC)/mass spectrometry (MS)-MS technology to analyze the biotinylated proteins. In total, 1351 biotinylated proteins were detected in HeLa cells and 1376 biotinylated proteins were found in HEK293T cells (Figure 2A, left panel). We then selected proteins that were enriched at least 10-fold (estimated by spectral counts) over the controls in both Hela and HEK293T cells, giving rise to 992 candidate proteins that were enriched in both cell types (Figure 2A, right panel; Table S1, related to Figure 2).

We performed cluster analysis with the 992 candidate proteins. First, going analysis of the cellular compartment revealed that most of the proteins were localized in intracellular membrane systems (Figure 2B). Interestingly, going analysis of biological process showed that these proteins mostly participated in cellular transport and localization (Figure 2C). A majority of Golgi-related proteins were among the biotinylated proteins surrounding PAQR3 (Figure 2D). These analyses, therefore, further supported our previous findings that PAQR3 is predominantly localized in the Golgi apparatus that is the central sorting station in the endomembrane system and plays important roles in endocytic trafficking (Glick and Nakano, 2009).

### PAQR3 Is Required for ER-to-Golgi Transport

**Figure 3:**
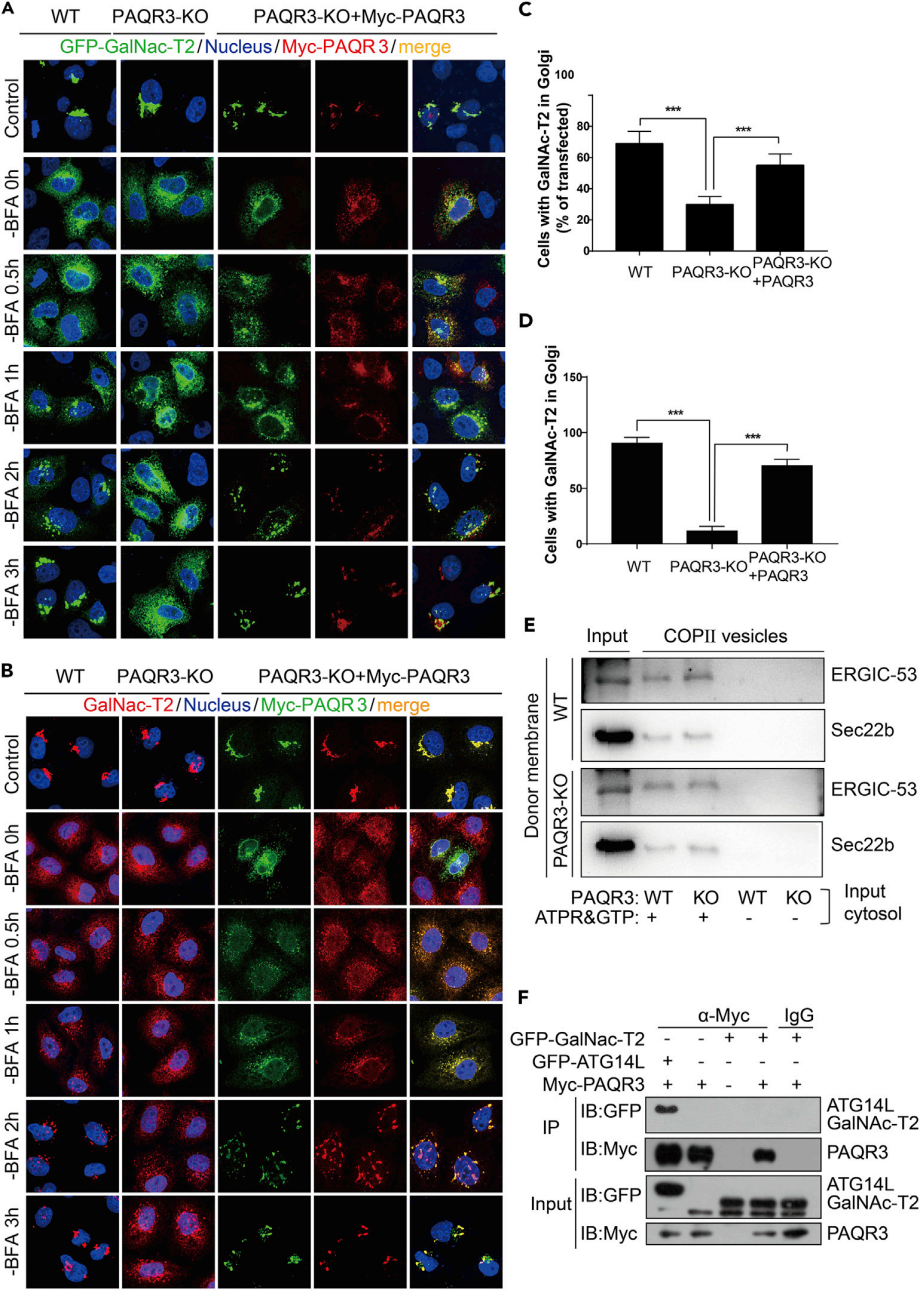
Deletion of PAQR3 Delays ER-to-Golgi Trafficking of GalNac-T2 (A and B) ER-to-Golgi transport of exogenous and endogenous GalNAc-T2 in HeLa cells. HeLa cells without or with PAQR3 deletion (WT or PAQR3-KO) were transiently transfected with GFP-GalNAc-T2 and Myc-tagged PAQR3 as indicated. At 24 hr after the transfection, the cells were treated with 5 μg/mL BFA for 30 min. The cells were then analyzed by fluorescence microscopy at different times after BFA removal. Endogenous GalNac-T2 was analyzed by an antibody against GalNAc-T2. The nucleus was stained with Hoechst 33342. All of the images were taken with the same exposure. (C and D) Quantitation of results of A and B. The quantitation of cells with GFP-fused GalNAc-T2 in the Golgi at 3 hr after BFA removal is shown in C. Quantitation of Golgi-localized endogenous GalNAc-T2 at 3 hr after BFA removal is shown in D. At least 500 cells were counted for the quantitation. The data are shown as mean ± SD, *** for p < 0.001. (E) Analysis of COPII vesicle budding *in vitro*. COPII-budding reactions using cytosolic fractions from WT or PAQR3-KO MEFs and membrane fractions (memb) from WT or PAQR3-KO MEFs. LMAN1/ERGIC-53 and Sec22b are COPII cargos, whose incorporation into budded vesicles needs energy supplied by ATP regeneration system (ATPR) and GTP. The experiment was repeated three times with similar results. (F) Analysis of interaction between PAQR3 and GalNAc-T2. Myc-tagged PAQR3, GFP-fused GalNAc-T2, and GFP-fused ATG14L were transiently expressed in HEK293T cells as indicated. After transfection for 24 hr, the cell lysate was used in immunoprecipitation (IP) and immunoblotting (IB) with the antibodies as indicated. ATG14L was used as a positive control (Xu et al., 2016). The experiment was repeated three times.

In most eukaryotic cells, the Golgi apparatus receives secretory cargoes exported from the ER and then sorts cargoes into a variety of transport carriers for delivery to their final destinations (Glick and Nakano, 2009). Previous research reports that Golgi-localized PAQR3 can control Golgi-to-PM protein transport via the Gβγ-PKD signaling pathway (Hewavitharana and Wedegaertner, 2015). We hypothesized that PAQR3 might also regulate ER-to-Golgi transport within a cell. To test this hypothesis, we analyzed the transport of N-acetylgalactosaminyltransferase-2 (GalNAc-T2), which was previously used as a model system to analyze ER-to-Golgi transport (Lippincott-Schwartz et al., 1989, Storrie et al., 1998). In brief, GalNAc-T2 is redistributed to ER in the presence of brefeldin A (BFA) and returns to the reformed Golgi apparatus upon BFA removal (Lippincott-Schwartz et al., 1989). A GFP-fused GalNAc-T2 (GalNAc-T2-GFP) was transiently expressed in both the wild type and PAQR3-deleted HeLa cells (Xu et al., 2016). At 30 min after BFA treatment, as expected, most of the GalNAc-T2-GFP was redistributed to ER (Figure 3A, the second vertical panel as compared with the first vertical panel). At 3 hr after BFA removal, ∼70% of the transfected cells had a clear Golgi localization of GalNAc-T2-GFP (Figures 3A and 3C). In contrast, only ∼30% of the transfected cells had GalNAc-T2-GFP returning back to the Golgi apparatus at this time point when PAQR3 was deleted (Figures 3A and 3C). We also performed a reconstitution experiment by overexpression of PAQR3 in the PAQR3-deleted cells. As shown in Figures 3A and 3B, most GalNAc-T2-GFP was redistributed to the Golgi apparatus on BFA removal in the presence PAQR3. We also analyzed ER-to-Golgi transport of endogenous GalNAc-T2. On BFA removal for 3 hr, almost all the GalNAc-T2 proteins were transported to the Golgi apparatus (Figure 3B). However, in PAQR3-deleted cells only ∼10% GalNAc-T2 proteins returned to the Golgi apparatus at this time point (Figures 3B and 3D). The endogenous GalNAc-T2-GFP protein was redistributed to the Golgi on BFA removal when PAQR3 was expressed in the PAQR3-deleted cells (Figures 3B and 3D). Furthermore, on BFA removal, reassembly of the Golgi apparatus was not affected by PAQR3 deletion (Figure S1, related to Figure 3), indicating that PAQR3 does not affect GalNAc-T2 trafficking through altering reassembly of Golgi ribbons. Collectively, these data indicated that PAQR3 has a positive effect on ER-to-Golgi transport of GalNAc-T2 as deficiency of PAQR3 drastically retarded the trafficking of GalNAc-T2 on removal of BFA.

The transport from ER to the Golgi is mediated by COPII-coated transport vesicles (Lord et al., 2013, Tang et al., 2005a, Watson and Stephens, 2005). In brief, the transport between ER and Golgi includes two major processes: budding of COPII-coated transport vesicles from ER and capture of the vesicles by cis-Golgi or ER-Golgi intermediate compartment (ERGIC) structures. We examined whether or not PAQR3 is involved in the budding process of COPII vesicles *in vitro*. The assay combined the cargo-containing membrane fractions from cell source with cytosolic extracts, which would supply soluble COPII proteins and putative regulatory proteins from another cell source (Joo et al., 2016). With this assay, we found that the cytosolic extracts derived from the wild-type and PAQR3-deleted mouse embryonic fibroblast (MEF) cells had no difference in COPII budding *in vitro* (Figure 3E). Taken together, these data indicated that PAQR3 is able to regulate ER-to-Golgi transport by a process independent of COPII budding. To rule out the possibility that PAQR3 might affect the trafficking of GalNAc-T2 by direct protein-protein interaction, we used a co-immunoprecipitation assay to investigate whether the two proteins could interact with each other. As shown in Figure 3F, PAQR3 could not interact with GalNAc-T2. However, as a positive control, PAQR3 could interact with ATG14L (Figure 3F), as previously reported (Xu et al., 2016).

**Figure 4:**
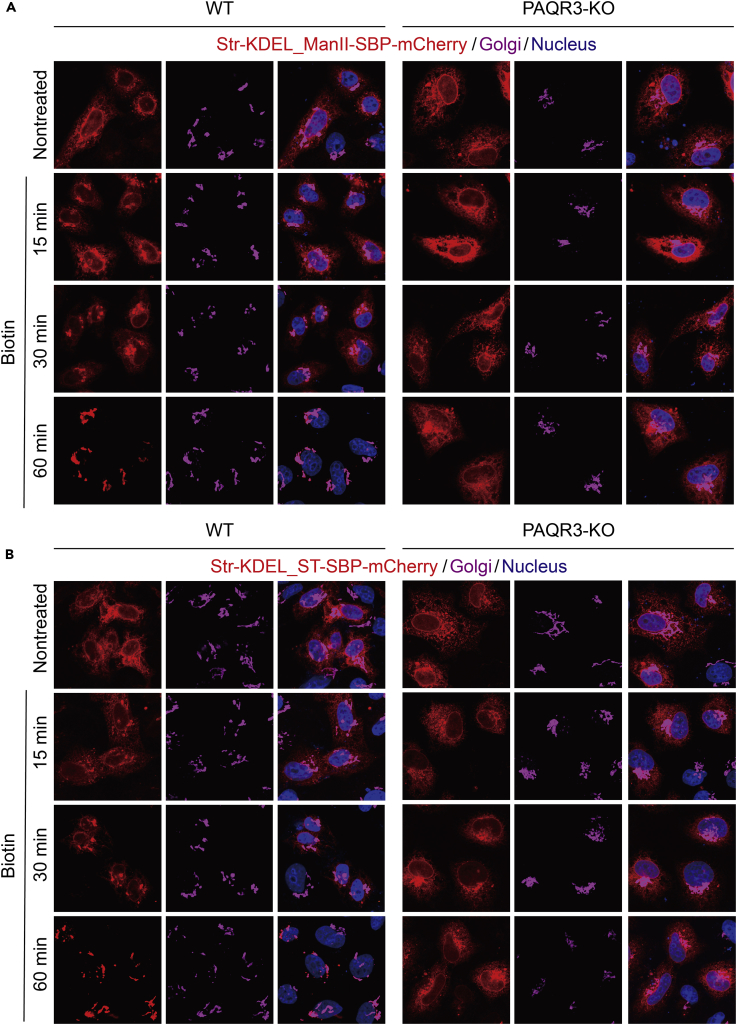
PAQR3 Deletion Reduces ER-to-Golgi Trafficking of Golgi-Reporter ST-SBP and ManII-SBP in RUSH Assay Wild-type HeLa cells (WT) or PAQR3-deficient HeLa cells (PAQR3-KO) were transiently transfected with Str-KDEL_ST-SBP-mCherry plasmid or Str-KDEL_ ManII -SBP-mCherry plasmid as indicated. About 36 hr after the transfection, 40 μM of biotin was added for different times. The cells were then analyzed by fluorescence microscopy. The Golgi was stained with antibody against GM130. The nucleus was stained with Hoechst 33342. All of the images were taken with the same exposure. The analysis was repeated three independent times.

To further investigate whether PAQR3 affects ER-to-Golgi transport, we applied another approach called the retention using selective hook (RUSH) assay (Boncompain et al., 2012). The RUSH assay is based on the reversible interaction of a streptavidin-fused protein (called “Hook”) stably anchored in the donor compartment with a streptavidin-binding peptide (SBP)-fused reporter protein (called “Reporter”). Biotin addition causes a synchronous release of the reporter from the hook. Streptavidin-fused KDEL (Str-KDEL) is an ER hook, whereas streptavidin-binding peptide-fused ST （ST-SBP） and α-mannosidase II (ManII-SBP) are Golgi reporters. ST-SBP and ManII-SBP were transiently co-expressed with Str-KDEL in both the wild-type and PAQR3-deleted HeLa cells. Before treatment with biotin, ST-SBP and ManII-SBP were anchored in the ER by Str-KDEL (Figure 4). On biotin addition for 60 min, ST-SBP and ManII-SBP trafficked to the Golgi (Figure 4). However, in PAQR3-deleted cells, ST-SBP and ManII-SBP failed to return to the Golgi at this time point (Figure 4). We then overexpressed exogenous PAQR3 in PAQR3-deleted cells and found that ST-SBP and ManII-SBP could be redistributed to the Golgi on biotin treatment (Figure S2, related to Figure 4). Therefore, these data further supported the notion that PAQR3 can modulate ER-to-Golgi transport.

### PAQR3 Interacts with COPII Coat Proteins Sec13 and Sec31A

**Figure 5:**
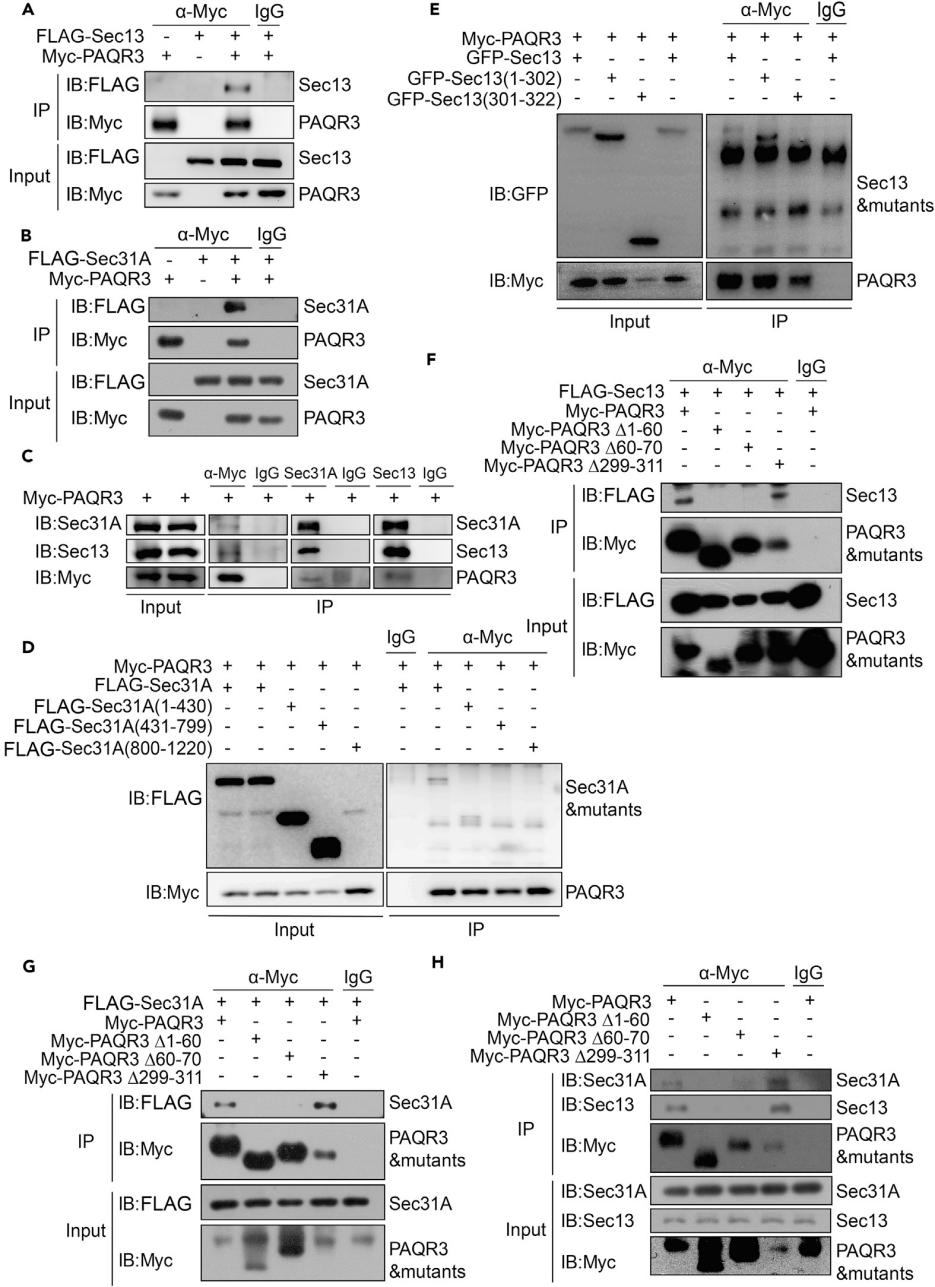
PAQR3 Interacts with COPII Components through its N-Terminal Region (A and B) Interaction of PAQR3 with ectopically expressed Sec13 and Sec31A. HEK293T cells were transiently transfected with Myc-tagged PAQR3, FLAG-tagged Sec13, and FLAG-tagged Sec31A as indicated. At 24 hr after the transfection, the cell lysate was used in immunoprecipitation (IP) and immunoblotting (IB) with the antibodies as indicated. (C) Interaction of PAQR3 with endogenous Sec13 and Sec31A. Myc-tagged PAQR3 was expressed in HEK293T cells. After transfection for 24 hr, the cell lysate was used in IP and IB with the antibodies as indicated. (D and E) Identification of the structural domain of Sec13 and Sec31A involved in the interaction with PAQR3. HEK293T cells were transiently transfected with PAQR3 and various mutants of Sec13 and Sec31A as indicated, followed by IP and IB analyses. (F–H) Identification of the domain of PAQR3 involved in the interaction with Sec13 and Sec31A. HEK293T cells were transiently transfected with various PAQR3 mutants and FLAG-tagged Sec13 or FLAG-tagged Sec31A as indicated. The cell lysate was analyzed by IP and IB with the antibodies as indicated. All the western blotting experiments were repeated at least twice with similar results.

As PAQR3 had no effect on COPII budding, we hypothesized that PAQR3 may act as an adaptor protein to tether COPII vesicle to the Golgi apparatus. The COPII complex includes two major heterodimeric coat proteins, the Sec23/Sec24 complex functioning as an inner shell and the Sec13/Sec31A complex functioning as an outer cage (Lord et al., 2013, Paccaud et al., 1996, Stagg et al., 2006). Intriguingly, all of these COPII coat proteins were found to be in close proximity to PAQR3 (Figure 2D). Structural analysis with these coat proteins indicated that both Sec31 and Sec13 contain WD domains. Our previous studies revealed that PAQR3 could interact with WD domains of many proteins (Jiang et al., 2010, Liu et al., 2015, Qiao et al., 2015). We therefore investigated whether or not PAQR3 could interact with Sec13 and Sec31A proteins. By co-immunoprecipitation assays, we found that Myc-tagged PAQR3 was able to interact with Flag-tagged Sec13 and Flag-tagged Sec31A (Figures 5A and 5B). Such observation was further supported by another co-immunoprecipitation assay in which we found that the Myc-tagged PAQR3 interacted with endogenous Sec13 and Sec31A proteins, respectively (Figure 5C).

We next investigated whether the WD domains of these two coat proteins are truly required for their interaction with PAQR3. The structure of Sec13 can be divided into two parts: the N-terminal WD40 repeat domain (1–302 amino acids) that is predicted to form a β-propeller (Saxena et al., 1996) and the C-terminal region (301–322 amino acids). The structure of Sec31A can be divided into three sections: the N-terminal WD40 repeat domain (1–430 amino acids), the intervening region (431–799 amino acids), and the C-terminal proline-rich region (800–1220 amino acids) (Yamasaki et al., 2006). Accordingly, we constructed various Sec13 and Sec31A mutants containing different domains and used them in co-immunoprecipitation assays. Only the WD40 repeat domains of Sec13 and Sec31A were able to interact with PAQR3, whereas the other domains could not (Figures 5D and 5E). Collectively, these data indicate that the WD domains of Sec13 and Sec31A are able to interact with PAQR3, thus potentially mediating the interaction of COPII vesicles with PAQR3.

We also investigated the structural domain of PAQR3 involved in the interaction by constructing a series of PAQR3 deletion mutants. By co-immunoprecipitation assays, we found that deletion of N-terminal 1–70 amino acids of PAQR3 led to loss of the binding capacity of PAQR3 with Sec13 and Sec31A (Figures 5F and 5G), consistent with our previous studies showing that the N-terminus of PAQR3 is required for Golgi localization and is important for its functions by interacting with multiple proteins (Luo et al., 2008, Xu et al., 2015, Xu et al., 2016, You et al., 2017, Zhao et al., 2018). Moreover, deletion of the N-terminal end of PAQR3 disrupted its interactions with endogenous Sec13 and Sec31A (Figure 5H), indicating that the N-terminal 1–70 amino acid residues of PAQR3 are required for the interaction of PAQR3 with these two COPII coat proteins.

We also used a glutathione S-transferase (GST) pull-down assay to determine whether the interaction of PAQR3 with the two COPII coat proteins and their WD domain was direct. The GST-fused N-terminal end of PAQR3 (1–71 aa), but not GST, could pull down Sec13, Sec31A, and their WD domains (Figure S3, related to Figure 5). Collectively, these data indicated that the N-terminus of PAQR3 is able to directly interact with these two COPII coat proteins through their WD domains.

### PAQR3 Tethers Sec13 and Sec31A to the Golgi Apparatus

**Figure 6:**
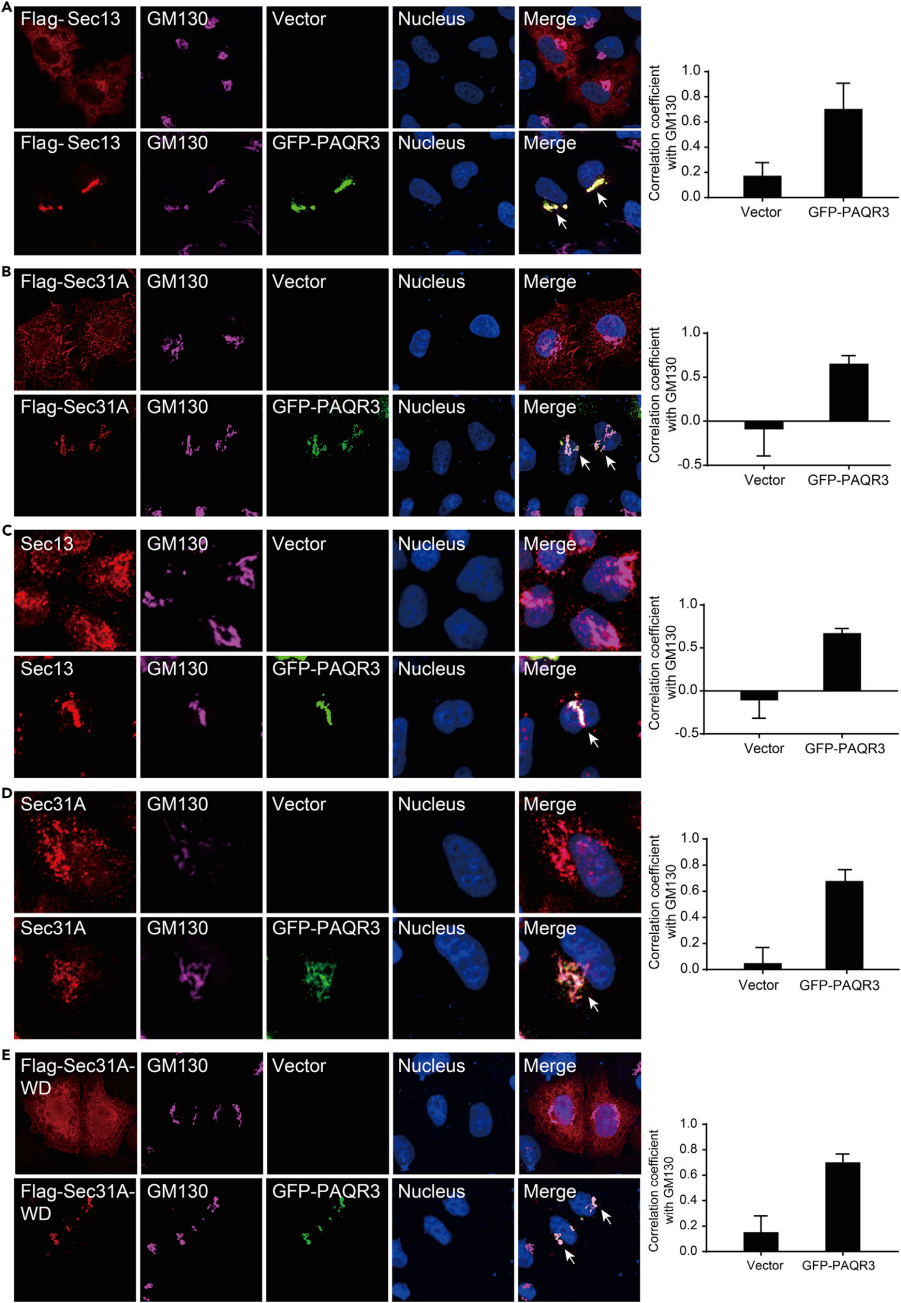
PAQR3 Tethers Sec13 and Sec31A to the Golgi Apparatus (A and B) Subcellular localization of exogenous Sec13 and Sec31A in the absence or presence of overexpressed PAQR3. HeLa cells were transiently transfected with FLAG-tagged Sec13, FLAG-tagged Sec31A, and GFP-fused PAQR3 as indicated. The transfected cells were fixed and then stained with an anti-FLAG antibody to detect Sec13/Sec31A. The Golgi was stained with an antibody against GM130, and the nucleus was stained with Hoechst 33342. The arrow indicates apparent co-localization of exogenous Sec13 and Sec31A with PAQR3. (C and D) Subcellular localization of endogenous Sec13 and Sec31A with or without the presence of PAQR3. HeLa cells were transfected with GFP-fused PAQR3 alone. The transfected cells were fixed and then stained with antibodies against Sec13 and Sec31A. The arrow indicates apparent co-localization of endogenous Sec13 and Sec31A with PAQR3. (E) Subcellular localization of the WD domain of Sec31A. HeLa cells were transiently co-transfected with FLAG-tagged WD domain of Sec31A (Sec31A-WD) and GFP-fused PAQR3. The transfected cells were fixed and then stained with an anti-FLAG antibody. The arrow indicates apparent co-localization of the WD domain of Sec31A with PAQR3. Quantitation of the co-localization of the proteins is shown in the right panel (n > 6).

When Sec13 and Sec31A were expressed alone in HeLa cells, both were mainly localized in the cytoplasm (Figures 6A and 6B), as previously reported (Enninga et al., 2003, Yamasaki et al., 2006). Consistently, endogenous Sec13 and Sec31A were diffusely distributed in the cytoplasm (Figures 6C and 6D), similar to previous reports (Helm et al., 2014, Townley et al., 2008). Interestingly, when Sec13 and Sec31A were co-expressed with PAQR3, most Sec13 and Sec31A proteins were localized in the Golgi apparatus (Figures 6A–6D). These observations are consistent with our previous finding that PAQR3 is mainly localized on the membrane of Golgi apparatus (Feng et al., 2007, Luo et al., 2008). Moreover, the distribution of endogenous Sec13 and Sec31A proteins was diverted to the Golgi apparatus by co-expressed PAQR3 protein (Figures 6C and 6D). In addition, the WD domain of Sec31A was diffusely districted in the cell (Figure 6E). However, this WD domain was localized in the Golgi apparatus when PAQR3 was co-expressed (Figure 6E). Taken together, these findings indicated that PAQR3 is able to tether Sec13 and Sec31A proteins to the Golgi apparatus, thus facilitating ER-to-Golgi transport of COPII vesicles.

### Mitochondria-Localized N Terminus of PAQR3 Tethers GalNAc-T2 and Sec22b to Mitochondria

**Figure 7:**
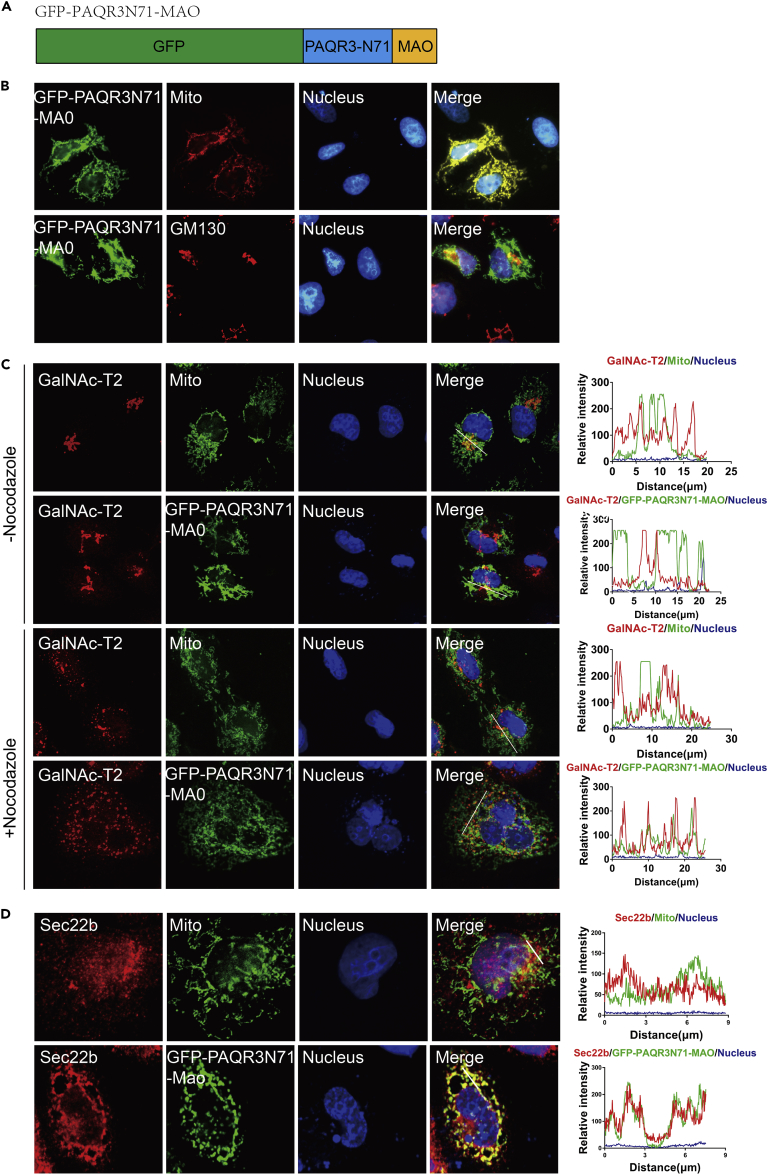
Mitochondria-Localized PAQR3 Alters Localization of GalNAc-T2 to the Mitochondria (A) A diagram depicting the construct of the N-terminal end (N71) of PAQR3 fused with GFP and the C-terminal transmembrane domain of MAO (GFP-PAQR3N71-MAO). (B) Subcellular distribution of GFP-PAQR3N71-MAO. HeLa cells were transiently transfected with GFP-PAQR3N71-MAO, followed by immunofluorescence staining. The mitochondria were stained by MitoTracker Deep Red FM, and the Golgi was stained with an antibody against GM130. The nucleus was stained with Hoechst 33342. (C) GFP-PAQR3N71-MAO changes localization of GalNAc-T2 to the mitochondria. HeLa cells expressing GFP-PAQR3N71-MAO were treated with nocodazole for 6 hr, and endogenous GalNAc-T2 was stained with a specific antibody. The mitochondria were stained by MitoTracker Deep Red FM, and the nucleus was stained with Hoechst 33342. Intensity plots of signal intensity (y axis) against distance in micrometers (x axis) are used to indicate occurrence of overlaps between the red and green channels. (D) GFP-PAQR3N71-MAO changes localization of Sec22b to the mitochondria. HeLa cells were transfected with GFP-PAQR3N71-MAO. Endogenous Sec22b was stained with a specific antibody. The mitochondria were stained by MitoTracker Deep Red FM, and the nuclei were stained with Hoechst 33342. Intensity plots of signal intensity (y axis) against distance in micrometers (x axis) are used to indicate occurrence of overlaps between the red and green channels.

To further consolidate our hypothesis that PAQR3 is able to tether COPII vesicles to the Golgi apparatus, we performed a “rerouting and capture assay” as reported previously (Wong and Munro, 2014). We fused the C-terminal transmembrane domain of monoamine oxidase (MAO) to the N-terminal 71 amino acid residues of PAQR3 (PAQR3N71) (Figure 7A). Consistent with the notion that the C-terminal transmembrane domain of MAO is sufficient for targeting to the outer mitochondrial membrane (Mitoma and Ito, 1992), PAQR3N71-MAO was clearly localized in the mitochondria but not in the Golgi apparatus (Figure 7B). GalNAc-T2 was mainly distributed in the Golgi apparatus with very limited overlap with mitochondrial marker or PAQR3N71-MAO (Figure 7C), consistent with a previous report showing that GalNAc-T2 is mainly accumulated in the Golgi stacks (Wong and Munro, 2014). To increase the probability for intracellular vesicles to encounter mitochondria, we used nocodazole to destroy microtubules and thus redistribute the Golgi into many ministacks throughout the cytoplasm as previously reported (Wong and Munro, 2014). Interestingly, nocodazole treatment caused robust overlaps of GalNAc-T2 with mitochondria-localized PAQR3N71-MAO (Figure 7C).

As the vesicles containing GALNAc-T2-GFP after nocodazole treatment could be dispersed Golgi vesicles or ERGIC vesicles but not COPII vesicles, we analyzed Sec22b, a marker for COPII vesicles in the absence of nocodazole. Sec22b itself was diffusely distributed in the cytoplasm without mitochondrial localization (Figure 7D). However, Sec22b was clearly co-localized with the mitochondria-localized PAQR3N71-MAO in the absence of nocodazole (Figure 7D). Thus, these observations further supported the hypothesis that PAQR3 can act as a Golgi tether protein to capture COPII vesicles released from ER.

### PAQR3 Is Localized in the ERGIC and *cis*-Golgi Structures

**Figure 8:**
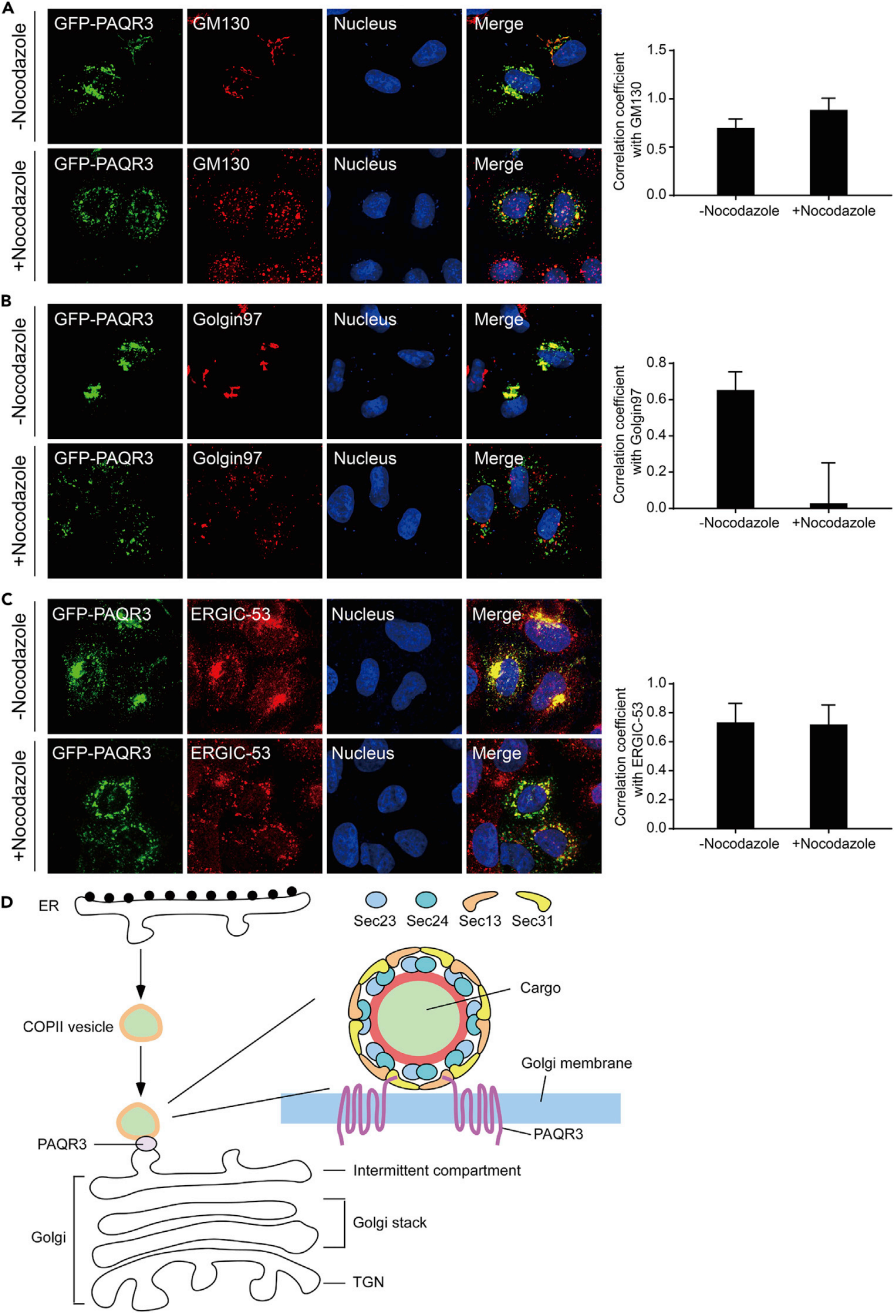
PAQR3 Is Localized in *cis*-Golgi and ERGIC Structures (A–C) Characterization of PAQR3 localization. HeLa cells were transiently transfected with GFP-fused PAQR3. After transfection for 24 hr, the cells were treated with or without nocodazole for 6 hr to destroy the Golgi, followed by immunofluorescence staining. The *cis*-Golgi was stained by an antibody against GM130 (A). The *trans*-Golgi was stained by antibody against Golgin97 (B). The ERGIC structure was stained with an antibody against ERGIC-53(C). The nucleus was stained with Hoechst 33342. Quantitation of the co-localization of the proteins is shown in the right panel (n > 6). (D) A model to depict the function of PAQR3 in regulating COPII trafficking. PAQR3 facilitates tethering of COPII vesicles to ERGIC/*cis*-Golgi through interaction with the Sec13/Sec31 coat proteins.

Newly synthesized secretory proteins are packaged into COPII vesicles at ER exit sites and then transported to the Golgi apparatus (Bannykh et al., 1996). The COPII vesicles can transport directly from ER to the Golgi in yeast (Cao et al., 1998, Lord et al., 2011). However, in mammalian cells, the COPII vesicles exit from ER and subsequently move to the ER-Golgi intermediate compartment (ERGIC) and *cis*-Golgi (Aridor et al., 1995, Farhan et al., 2008). The ERGIC is a complex membrane system between ER and Golgi with two main functions: to tether cargo from the ER and to sort cargo for onward anterograde transport or retrograde retrieval back to the ER (Lord et al., 2013, Martinez-Menarguez et al., 1999). The marker of ERGIC is the lectin ERGIC-53 (Appenzeller-Herzog and Hauri, 2006). As we proposed that PAQR3 acts as a tether for COPII vesicles, it is necessary to address whether or not PAQR3 is localized in the COPII acceptor structure of the Golgi. We expressed GFP-fused PAQR3 in HeLa cells and then used nocodazole to destroy Golgi. Next, we applied immunofluorescent staining to visualize the localization of PAQR3 with markers of *cis*-Golgi, *trans*-Golgi, and ERGIC. GM130 and Golgin97 were used to mark *cis*- and *trans*-Golgi, respectively, whereas ERGIC-53 was used to mark ERGIC. Clearly, PAQR3 was localized in the *cis*-Golgi and ERGIC but not in the *trans*-Golgi (Figures 8A–8C). In particular, the PAQR3 puncta around the Golgi were almost completely co-localized with ERGIC-53 (Figure 8C). Collectively, we propose that PAQR3 is able to facilitate trafficking of COPII vesicles from ER to ERGIC or *cis*-Golgi via interaction with Sec13/Sec31 complex (Figure 8D). At the molecular level, PAQR3 promotes tethering of COPII vesicles to the Golgi via interaction of its N-terminal end with the WD domains of Sec13 and Sec31 coat proteins (Figure 8D).

## Discussion

Our finding in this study is in exquisite agreement with the function of PAQR3 in regulating Scap/SREBP2 transport from ER to Golgi to control homeostasis (Xu et al., 2015). Cellular cholesterol level is regulated by a negative feedback determined by subcellular localization of SREBP2, a critical transcription factor dictating expression of an array of genes required for *de novo* synthesis of cholesterol (Brown and Goldstein, 1997). When the cellular cholesterol level is high, SREBP2, together with its binding partner Scap, is retained in ER via interaction with the ER-localized INSIG protein (Brown et al., 2002). Low level of cellular cholesterol levels leads to ubiquitination and degradation of INSIG, leading to trafficking of Scap/SREBP2 complex from ER to Golgi via COPII vesicles (Gong et al., 2006, Sun et al., 2005). Previous studies have revealed that PAQR3 acts an anchor protein in the Golgi to facilitate tethering of Scap/SREBP2 complex to the Golgi, thus promoting protease-mediated processing and activation of SREBP2 (Xu et al., 2015). Thus, we postulate that PAQR3 fulfills this function to control cholesterol homeostasis by tethering COPII vesicles to the Golgi as demonstrated in this study.

It remains a question whether or not PAQR3 can choose specific COPII vesicles in ER-to-Golgi trafficking. The study with the Scap/SREBP2 complex indicates that PAQR3 can interact with certain regions of Scap and SREBP2 (Xu et al., 2015). It is therefore possible that PAQR3 has a preference to certain cargos of COPII vesicle in addition to its binding to Sec13/Sec31 coat proteins. If this were the case, it will be of interest to identify the spectrum of cargo proteins that are facilitated by PAQR3 during ER-to-Golgi transport. In addition, another unaddressed question is whether or not PAQR3 is involved in the trafficking of COPI vesicles, which are required for retrograde transport from ERGIC to ER as well as anterograde transport from ERGIC to the Golgi (Rothman and Wieland, 1996, Spang, 2013). Interestingly, the APEX experiment revealed that many COPI coat proteins were identified in close proximity to PAQR3 (Figure 2). Furthermore, some of these COPI coat proteins, such as COPA and COPB2, contained a WD40 repeat domain, which has been found to be a structural motif interacting with PAQR3 (Jiang et al., 2010, Wang et al., 2017, Xu et al., 2015). Thus, we predict that the Golgi-localized PAQR3 could interact with COPI vesicles and aid their tethering to the Golgi. This is an intriguing question necessary to be addressed in the future.

PAQR3 mainly functions as a tumor suppressor that has an inhibitory function in many types of tumors (Jiang et al., 2011, Ling et al., 2014, Wang et al., 2012, Xie et al., 2008, Zhang et al., 2010). It is currently unknown whether the tumor suppressive activity of PAQR3 is associated with its regulatory function on ER-to-Golgi trafficking. Considering that a well-balanced and controlled intracellular trafficking is crucial in maintaining cellular homeostasis, we speculate that downregulation of PAQR3 found in many types of tumors would be associated with dysregulation of cellular homeostasis as a result of deregulated ER-to-Golgi transport. This notion is in agreement with the findings that intracellular trafficking is closely associated with many human disorders (Izumi et al., 2016, Khoriaty et al., 2012, Routledge et al., 2010). Therefore, our finding that PAQR3 plays an important role in the transport of COPII vesicles not only expands our comprehension about the complexity of intracellular trafficking but may also increase our understanding of the molecular basis of human diseases.

### Limitations of the Study

There are a few limitations of the current study. One of them is vesicle selectivity. Although our study reveals that PAQR3 can facilitate ER-to-Golgi trafficking of COPII vesicles, it is unknown whether PAQR3 has selectivity for certain types of vesicles. One possibility is that PAQR3 may also interact with cargo proteins to aid in choosing certain types of vesicles. For example, a previous study has shown that PAQR3 can interact with the Scap/SREBP complex to help tethering them to the Golgi and this complex is transported from ER to the Golgi via COPII vesicles (Xu et al., 2015). Another limitation of the study is that it has not addressed whether or not PAQR3 is involved in the regulation of trafficking of vesicles other than COPII. As PAQR3 can interact with WD domain-containing proteins and other vesicle coat proteins such as those of COPI vesicles also contain WD domains, it will be of interest to investigate if PAQR3 can also modulate the transport of COPI vesicles and others. In addition, this study lacks dynamic analysis to track the ER-to-Golgi trafficking of COPII vesicles in real time. Analysis with high-resolution microscopy is needed to provide live images to profile the dynamics of vesicle tethering by PAQR3.

## Methods

All methods can be found in the accompanying Transparent Methods supplemental file.

## Supplemental Information

Document S1. Transparent Methods and Figures S1–S3

Table S1. The List of Proteins Identified by the Proximity Labeling Assay, Related to Figure 2 The list of 992 proteins that were enriched at least 10-fold (estimated by spectral counts) over the controls in both HeLa and HEK293T cells by the proximity labeling assay.
