# Supplementary material for: PAQR3 Regulates Endoplasmic Reticulum-to-Golgi Trafficking of COPII Vesicle via Interaction with Sec13/Sec31 Coat Proteins
Source: iScience. 2018 Nov 4;9:382–98. doi: 10.1016/j.isci.2018.11.002 (PMC6249397; doi:10.1016/j.isci.2018.11.002)
Supplement: Document S1. Transparent Methods and Figures S1–S3 [file mmc1.pdf]

ISCI, Volume *9*

Supplemental Information

PAQR3 Regulates Endoplasmic Reticulum-to-Golgi Trafficking of COPII Vesicle via Interaction with Sec13/Sec31 Coat Proteins

Qianqian Cao, Zheng Wang, Huida Wan, Lijiao Xu, Xue You, Lujian Liao, and Yan Chen

Transparent Methods and Supplemental Figures

Transparent Methods

Materials and antibodies

Materials used in this study include: Nocodazole (Beyotime Biotechnology, ShangHai, China); Brefeldin A (MedChemExpress, New Jersey, USA); MitoTracker® Deep Red CM-H2Ros (Invitrogen, Frederick, MD, USA); Streptavidin magnetic beads (Thermo Scientific, Waltham, MA, USA); NeutrAvidin biotin-binding protein (Invitrogen); Alexa Fluor 647 succinimidyl ester (Invitrogen); Biotin-phenol (Toronto Research Chemicals, Ontario, Canada); Biotin (Sigma-Aldrich). The antibodies used in this study included human Sec13 (A11613, ABclonal, Boston, USA); human Sec31A (A9321, ABclonal); human GalNAc-T2 (A6910, ABclonal); human ERGIC-53 (sc-66880, Santa Cruz Biotechnology, Santa Cruz, CA); human Sec22b (sc-101267, Santa Cruz Biotechnology); Myc tag (sc-40, Santa Cruz Biotechnology); GFP tag (sc-9996, Santa Cruz Biotechnology); Flag tag (F3165, Sigma-Aldrich); GM130 (ab52649, Abcam, Cambridge, UK); Golgin97(A21270, Life Technologies, Carlsbad, CA, USA); Streptavidin-horseradish peroxidase (SA-HRP, S911, Life Technologies).

Cell culture and transfection

HEK293T and HeLa cell lines were cultured in DMEM (Invitrogen) medium containing 10% fetal bovine serum (FBS; Invitrogen) and 50 mg/ml penicillin/streptomycin at 37 °C with 5% CO_2. Establishment of PAQR3-deficient HeLa cells was described as previously reported (Xu et al., 2016). The stable Sec31A knock-down Hela cell lines were generated by lentivirus infection as previous described (Xu et al., 2016)**.** Short hairpin RNA (shRNA) constructs for Sec31A were inserted into the pLenti hU6-MCS-CMV-Puro vector (LncBio Company, ShangHai, China). The annealed shRNA cassette targets sequences of 5’-AACAGGAACATCTGCTCAGCAAT-3’ and 5’- AATTGCTTGGAGCATGGCAGATC -

3’ for human Sec31A. Transient transfection in HEK293T and HeLa cell lines was performed by polyethylenimine (Sigma-Aldrich) and Polyjet (SignaGen, Rockville, USA) transfection reagents respectively.

Plasmid construction

The Myc-tagged and GFP-tagged PAQR3 were described previously (Feng et al., 2007). Full-length coding Sec13 and Sec31A were cloned by RT-PCR from human cells and confirmed by DNA sequencing. All deletion mutants of PAQR3 and Sec31A were subcloned into the corresponding vectors and confirmed by DNA sequencing. GFP-fused GalNAc-T2 was purchased from Sino Biological (Beijing, China). To construct APEX2-fused PAQR3, we purchased APEX2-actin from Addgene and replaced actin with Flag-tagged human PAQR3 by using restriction enzymes XhoI/BamHI (TaKaRa, Dalian, China). The detailed construction of GFP-PAQR3N71-MAO was following to a previous report (Wong and Munro, 2014). Briefly, the N-terminal of PAQR3 (1-71 amino acids, PAQR3N71) and the C-terminal transmembrane domain of monoamine oxidase (MAO) were fused and then cloned into EGFP-C1 vector to relocate PAQR3N71 to mitochondrial membranes.

APEX-labeling experiment

The experiment was performed as previously reported (Hung et al., 2016). In brief, HEK293T and Hela cells were transfected with indicated APEX2-PAQR3 plasmid. About 24 h after transfection, the cells were incubated in the presence of 0.5 mM biotin-phenol for 30 min, after which hydrogen peroxide (H_2O_2) was added to a final concentration of 1mM, followed by incubation at room temperature for 1 min. The labeling solution was quickly aspirated and the cells were washed three times in quencher solution (10 mM sodium ascorbate, 5 mM Trolox and 10 mM sodium azide solution in PBS). For microscopic analysis, cells were immediately fixed with 4% formaldehyde. For Western

blotting analyses, the cells were lysed in RIPA lysis buffer (50 mM Tris/HCl, pH 7.5, 150 mM NaCl, 0.1%SDS, 0.5% sodium deoxycholate and 1% Triton X-100) supplemented with 1X protease inhibitor cocktail , 1 mM PMSF and quenchers (10 mM sodium azide, 10 mM sodium ascorbate and 5 mM Trolox). The cell lysate was cleared by centrifuging at 15,000g for 10 min at 4°C and the supernatant was used immediately for SDS-PAGE or stored at -80°C.

Streptavidin chromatography

Protein concentrations of lysates prepared from APEX-labeling experiments were determined by Bradford assay and the lysates were adjusted to equal protein concentrations. Then the following experiment was performed according to a previous report (Hung et al., 2016). For each sample, 10% of the sample was taken as input and the remaining was incubated with streptavidin magnetic beads for 1 h at room temperature on a rotator. The beads were pelleted with a magnetic rack and the supernatant was collected (flow-through). The beads were washed with a series of buffers (1 ml for each wash) to remove nonspecific binders: twice with RIPA lysis buffer, once with 1 M KCl, once with 0.1 M Na_2CO_3, once with 2 M urea in 10 mM Tris/HCl, pH 8.0, and twice with RIPA lysis buffer. Eluted biotinylated proteins from the beads by boiling each sample in protein loading buffer supplemented with 2 mM biotin and 20 mM DTT for 10 min. The samples were placed on a magnetic rack to pellet the beads and to collect the eluate for further analyses.

LC−MS/MS analysis

The experiment was performed as previously report (Xu et al., 2016). In brief, the purified biotinylated proteins were resuspended and reduced in lysis buffer (8 M urea, 100 mM TEAB, 1 mM sodium orthoranadate, 5 mM sodium fluoride, 5 mM β-glycerophosphate, 10 mM DTT, pH 8.0), and then alkylated with 50 mM iodoacetamide. After reducing urea concentration to 2 M, the samples were digested with trypsin (Promega, Madison,

Wisconsin, USA) at 37°C (1:100 w/w) overnight. The digested peptide mixtures were acidified with trifluoroacetic acid (TFA) at 1% final concentration and desalted by reversed-phase C18 Sep-Pak cartridge (Millipore, MA, USA). Peptides were loaded and separated on an EASY-nLC1000 LC (Thermo Scientific) coupled on line to the Q-Exactive Orbitrap mass spectrometer (Thermo Scientific). The analytical column was constructed in a 150 mm×75 μm silica capillary tip pulled with a column puller and packed with 3-μm Luna C18 stationary phase. Peptides were eluted with a 120 min gradient from 12 to 32% buffer B (98% ACN, 0.1% formic acid) at a flow rate of 250 nL/min. Full MS were acquired at a resolution of 70,000 with an AGC target value of 3×10^6 and a maximum injection time of 100 ms. Target value for the MS/MS scans was 1×10^5 charges with a resolution of 17,500 and a maximum injection time of 50 ms. The raw files were processed by MaxQuant software (version 1.5.3.8). For the database searching parameters, the precursor mass tolerance was set to 10 ppm. Trypsin/P was set as the protease, accounting for in-source fragmentation of lysine or arginine residues, followed by proline. Two missed cleavages were allowed and all data were searching against the UniProt Human database.

Co-immunoprecipitation and immunoblotting

For co-immunoprecipitation (Co-IP) assays, the transfected HEK293T cells were washed once with ice-cold PBS, then lysed with ice-cold lysis buffer (10 mM Tris/HCl, pH7.5, 2 mM EDTA, 150 mM NaCl, 1% NP-40, 50mM NaF, 1 mM Na_3VO_4 , and protease inhibitor cocktail (Roche) for 30 min at 4°C. The homogenates were centrifuged for 15 min at 12,000 rpm at 4°C. 10% of the supernatant was harvested for Western blotting analysis as inputs, while the remaining cell lysate was incubated with primary antibodies overnight at 4°C. Protein A/G plus agarose (Genescript, New Jersey, USA) was added at 4°C for 2 h. The resulting beads were washed with lysis buffer for 5 times, followed by Western blotting analysis.

Immunofluorescence staining and protein co-localization quantitation

The methods for immunofluorescence staining were described previously (You et al., 2017). NeutrAvidin-fluorophore conjugate (conjugate NeutrAvidin biotin-binding protein and Alexa Fluor 647 succinimidyl ester as Invitrogen's instructions) was used to mark biotinylated proteins in cells. Images were acquired with a ZEISS LSM880 confocal microscope using a 63X Apochromat oil-immersion objective. Images processing and quantification of fluorescence intensity was performed with ZEISS ZEN 2.3 lite software. Co-localization was quantified by calculating Pearson's correlation coefficient value using co-localization plugin of ZEN.

Glutathione S-transferase (GST) pulldown assay

The N-terminal 1–71 amino acid of PAQR3 was fused with PGEX-4T-1 vector, while Sec13, Sec31A and their WD Domain were fused with a His-containing vector (PET-28a). The GST pulldown assay was performed as described previously (Xu et al., 2016).

RUSH assay

The RUSH assay was performed as previous report (Boncompain et al., 2012). Str-KDEL_ST-SBP-mCherry and Str-KDEL_ManII-SBP-mCherry were purchased from Addgene. Hela cell lines were transiently transfected with Str-KDEL_ST-SBP-mCherry or Str-KDEL_ManII-SBP-mCherry. About 36h after transfection, 40 μM of biotin was added for 0 min, 15 min, 30 min or 60 min at 37°C and cells were then fixed for Immunofluorescence staining.

COPII vesicle formation Assay

The membrane fractions of wild type MEF and PAQR3-deleted MEF cells cells were used as a source of membrane and cargo, while the cytosol of wild type MEF cells and PAQR3-deleted MEF cells were used as a source of soluble COPII proteins. The detailed steps of

vesicle-budding reaction were described as previous reported (Ge et al., 2013; Kim et al., 2005).

Statistical analyses

Student's t-test was used for all the statistical analyses. The p value < 0.5 was considered statistically significant and all results were shown as mean ± standard deviation (SD).

References

Boncompain, G., Divoux, S., Gareil, N., de Forges, H., Lescure, A., Latreche, L., Mercanti, V., Jollivet, F., Raposo, G., and Perez, F. (2012). Synchronization of secretory protein traffic in populations of cells. Nat Methods *9*, 493-498.

Feng, L., Xie, X.D., Ding, Q.R., Luo, X.L., He, J., Fan, F.J., Liu, W.Z., Wang, Z.Z., and Chen, Y. (2007). Spatial regulation of Raf kinase signaling by RKTG. Proc Natl Acad Sci USA *104*, 14348-14353.

Ge, L., Melville, D., Zhang, M., and Schekman, R. (2013). The ER-Golgi intermediate compartment is a key membrane source for the LC3 lipidation step of autophagosome biogenesis. Elife *2*.

Hung, V., Udeshi, N.D., Lam, S.S., Loh, K.H., Cox, K.J., Pedram, K., Carr, S.A., and Ting, A.Y. (2016). Spatially resolved proteomic mapping in living cells with the engineered peroxidase APEX2. Nat Protocols *11*, 456-475.

Kim, J., Hamamoto, S., Ravazzola, M., Orci, L., and Schekman, R. (2005). Uncoupled packaging of amyloid precursor protein and presenilin 1 into coat protein complex II vesicles. J Biol Chem *280*, 7758-7768.

Wong, M., and Munro, S. (2014). The specificity of vesicle traffic to the Golgi is encoded

in the golgin coiled-coil proteins. Science *346*, 601-+.

Xu, D.Q., Wang, Z., Wang, C.Y., Zhang, D.Y., Wan, H.D., Zhao, Z.L., Gu, J., Zhang, Y.X., Li, Z.G., Man, K.Y., *et al.* (2016). PAQR3 controls autophagy by integrating AMPK signaling to enhance ATG14L-associated PI3K activity. Embo J *35*, 496-514.

You, X., Guo, W., Wang, L., Hou, Y., Zhang, H., Pan, Y., Han, R., Huang, M., Liao, L., and Chen, Y. (2017). Subcellular distribution of RAD23B controls XPC degradation and DNA damage repair in response to chemotherapy drugs. Cell Signal *36*, 108-116.

Supplemental Figures

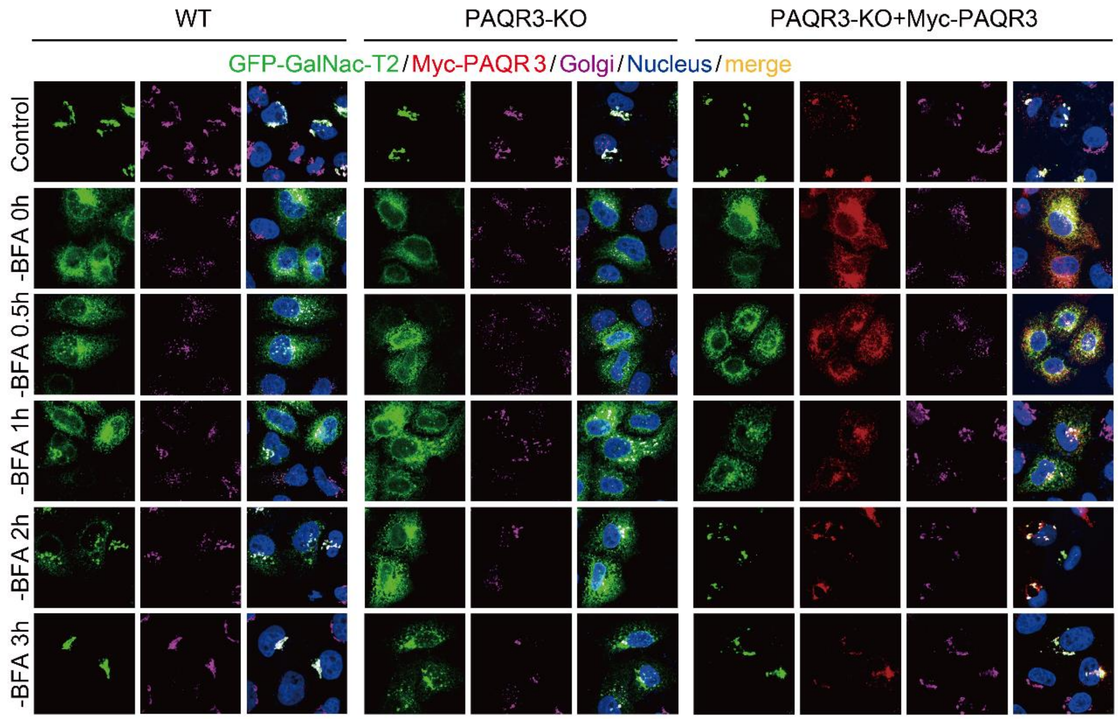

Figure S1. PAQR3 deletion has no effect on Golgi ribbon reassembly, Related to Figure 3

Wildtype Hela cells (WT) or PAQR3-deficient Hela cells (PAQR3-KO) were transiently transfected with GFP-GalNAc-T2 and Myc-tagged PAQR3 as indicated. About 24 h after the transfection, the cells were treated with 5 μg/ml BFA for 30 min. The cells were then analyzed by fluorescence microscopy at different times after BFA removal. PAQR3 was stained with antibody against Myc tag. Golgi was stained with antibody against GM130. The nucleus was stained with Hoechst 33342. All of the images were taken with the same exposure.

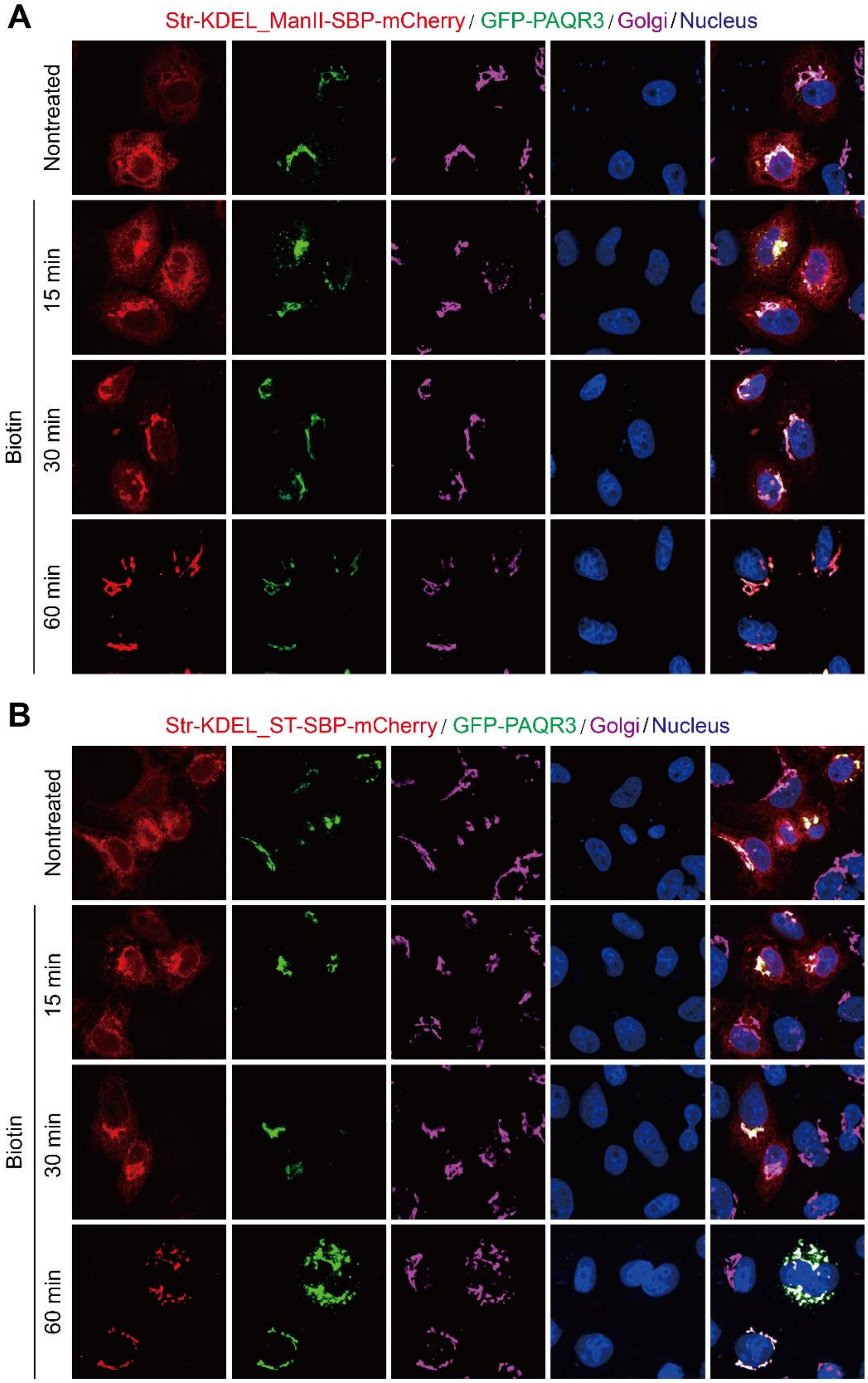

Figure S2. Overexpression of PAQR3 in PAQR3-deficient Hela cells rescues ER-to-Golgi trafficking in RUSH assay, Related to Figure 4

ST-SBP and ManII-SBP return to the Golgi apparatus in the presence of PAQR3 in PAQR3-deficient Hela cells. PAQR3-deficient Hela cells (PAQR3-KO) were transiently co-

transfected with Myc-tagged PAQR3 and Str-KDEL_ST-SBP-mCherry or Str-KDEL_ ManII -SBP-mCherry as indicated. Then the transfected cells were treated with biotin for indicated times and analyzed by fluorescence microscopy. PAQR3 was stained with antibody against Myc tag. Golgi was stained with antibody against GM130. The nucleus was stained with Hoechst 33342. All of the images were taken with the same exposure.

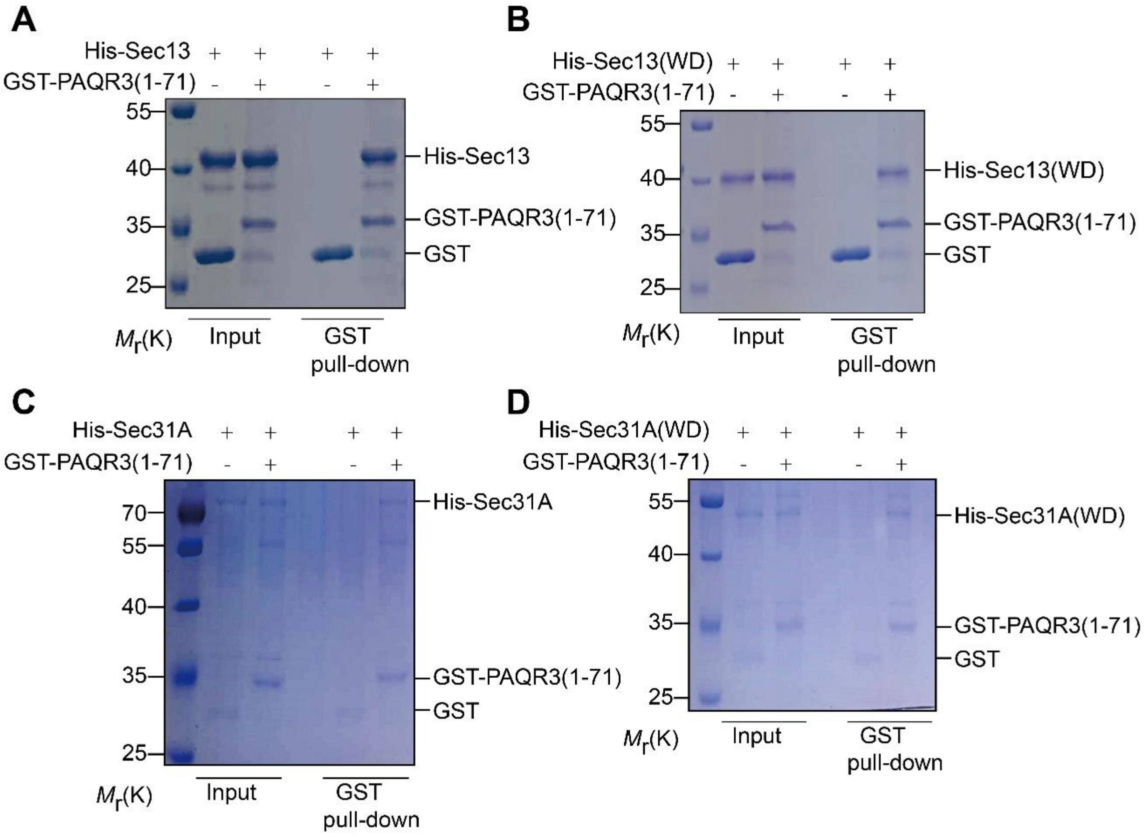

Figure S3. PAQR3 has a direct interaction with Sec13/Sec31A, Related to Figure 5

(A) Direct interaction of PAQR3 with Sec13 *in vitro*. GST-fused N-terminal 1–71 aa of PAQR3 and His-fused Sec13 were used in a GST-pulldown assay.

(B) Direct interaction of PAQR3 with the WD domain of Sec13 *in vitro*. GST-fused N-terminal 1–71 aa of PAQR3 and His-fused WD domain of Sec13 were used in a GST-pulldown assay.

(C) Direct interaction of PAQR3 with Sec31A *in vitro.* GST-fused N-terminal 1–71 aa of PAQR3 and His-fused Sec31A were used in a GST-pulldown assay.

(D) Direct interaction of PAQR3 with the WD domain of Sec31A *in vitro*. GST-fused N-terminal 1–71 aa of PAQR3 and His-fused WD domain of Sec31A were used in a GST-pulldown assay.
